# Incidentally Diagnosed Multiple Vascular Lesions of the Spleen: Littoral Cell Angioma or Hemangioma?

**DOI:** 10.21699/ajcr.v7i5.492

**Published:** 2016-11-01

**Authors:** Emrah Aydin

**Affiliations:** Pediatric Surgery, Bahcelievler State Hospital, Bagcilar Education and Training Hospital, Istanbul, Turkey

**Keywords:** Spleen, Hemangioma, Littoral cell Angioma, Vascular lesion

## Abstract

Vascular lesions of the solid abdominal viscera may pose diagnostic and management issues. A 16-year old girl admitted to emergency department due to recurrent abdominal pain and diagnosed to have multiple vascular malformations of the spleen on imaging investigations. Littoral cell angioma was preoperative suspicion owing to no response of the vascular lesion to the propranolol. It turned out to be cavernous hemangioma on histopathology.

## INTRODUCTION

Primary tumors of the spleen are classified as lymphoid tumors, non-hematolymphoid tumors and tumor like lesions. Vascular tumors of spleen are rare lesions and include hemangioma, hamartoma, lymphangioma, Littoral cell angioma, and angiosarcoma. Littoral cell angioma was first described by Falk et al in 1991. It arises from the lining cells of the red-pulp sinuses.[1] Splenectomy has to be done for final diagnosis. Hemangioma is the most common benign neoplasm of the spleen. These are small in size and incidentally diagnosed. Preoperative diagnosis is difficult if these are multiple in number with cavernous characteristic.[1-4] Herein, we present a case operated due to suspicion for Littoral cell angioma but diagnosed as multiple cavernous hemangioma on histopathology.

## CASE REPORT

A 16-year old girl was admitted to emergency department due to recurrent epigastric pain for the last six months. Her general physical examination was normal. Abdominal examination was also unremarkable. Laboratory investigations were within normal limits except for mild hyperbilirubinemia. Abdominal ultrasonography revealed multiple vascular malformations of the spleen largest being 15mm in diameter. A computerized tomography with oral and intravenous contrast showed thickening of gastric and intestinal walls, multiple enlarged paraaortic and parailiac lymph nodes; spleen showed 11 hypodense lesions, largest being 17mm in diameter. On MRI, splenic lesions were hypointense at T1 and hyperintense at T2 with minute peripheral contrast enhancement (Fig.1). Preoperative suspicion was of hemangioma and lymphoma (given the findings of gastric and intestinal wall thickening and enlarged lymph nodes). She was consulted with oncology department for suspected malignancy and gastroenterology department for suspected Gilbert syndrome but ruled out on investigations. Upper and lower gastrointestinal endoscopy were performed and biopsies were taken that did not show any pathology. Beta blocker treatment was started with proton pump inhibitors but no improvement was seen on repeat MRI. Splenectomy was then performed with a suspicion of Littoral cell angioma as an alternate diagnosis. Grossly, the spleen had nodular spongy dark lesions, ranging 1 to 2cm in diameter (Fig.2). On histopathological examination, it was reported as multiple cavernous hemangioma. She remained well at the last follow up six months back. 

**Figure:**
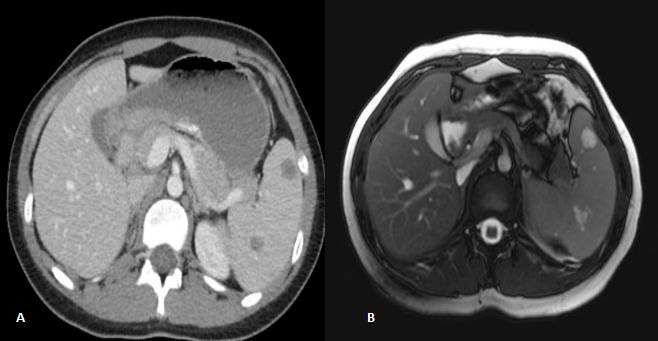
Figure 1:Computerized tomography (A) and magnetic resonance imaging (B) of the patient.

**Figure:**
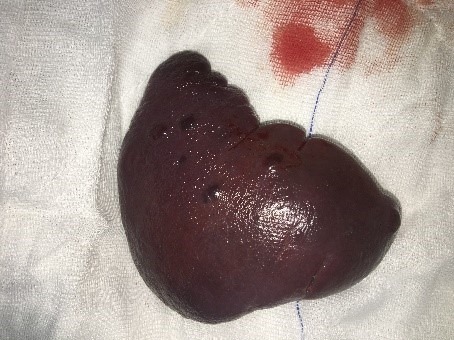
Figure 2:Per operative view of the spleen with lesions.

## DISCUSSION

Vascular tumors are the most common neoplasms of the spleen. They arise form vascular elements i.e. the red pulp of the spleen. Signs and symptoms may vary from being asymptomatic to splenic rupture or hemorrhage.[1] Diagnosis is supported by preoperative workup, especially imaging investigations such as CT scan and MRI, but is quite challenging. It has no specific clinical features. Our patient presented with recurrent episodes of abdominal pain and diagnosed to have vascular lesions of the spleen as an incidental finding on USG.

Ultrasound findings of Littoral cell angioma are nonspecific. On ultrasound it can vary from cystic, hypoechoic mass to a homogenously hyperechoic mass. On CT scan, it is iso-dense or slightly hyperdense (without contrast); with contrast it is hypodense on arterial phase with heterogeneous to homogenous enhancement on venous phase which is again nonspecific. MRI findings of Littoral cell angioma may differ depending on the amount of siderosis which is highly variable.

Ultrasound features of hemangioma is a well-defined intrasplenic or pedunculated echogenic solid or complex cystic mass. At computerized tomography, cavernous hemangiomas appear as isodense or hyperdense due to its solid component. Splenic hemangiomas are hypo to isointense when compared with normal spleen at magnetic resonance imaging.[2] In our case, all radiologic work up was non-specific. We could only say that it was a vascular tumor of the spleen. More interestingly thickening of the wall of stomach and intestine were found at computerized tomography which also favored Littoral cell angioma as almost half of the cases have immunosuppression or malignancy. The other possibility was of lymphoma. The laboratory analysis of the patient revealed only mild hyperbilirubinemia which remained for three months. That’s why we also thought of Gilbert’s syndrome but no pathology found at specific work up.

Because hemangioma is more frequent than Littoral cell angioma we preferred medical treatment with beta blocker drugs. In case the lesion is hemangioma, it mostly shrinks in few months after the treatment. In our case, three months medical therapy did not reduce the size or number of the lesions therefore Littoral cell angioma became our second differential. Littoral cell angioma can be managed with chemotherapy, partial splenectomy or splenectomy.[4] In our case, splenectomy was the only choice due to multiple lesions involving the entire spleen and moreover true nature of the lesions was also in question. Vascular lesions of the spleen though rare may sometimes become challenging to diagnose.

## Footnotes

**Source of Support:** Nil

**Conflict of Interest:** None declared

